# *Candidatus* Mycoplasma haemohominis in Human, Japan

**DOI:** 10.3201/eid2601.190983

**Published:** 2020-01

**Authors:** Norimichi Hattori, Makoto Kuroda, Harutaka Katano, Takahiro Takuma, Takayoshi Ito, Nana Arai, Ryo Yanai, Tsuyoshi Sekizuka, Sho Ishii, Yoko Miura, Takahiro Tokunaga, Hiroyuki Watanabe, Norihiro Nomura, Junichi Eguchi, Hideki Hasegawa, Tsuyoshi Nakamaki, Takaji Wakita, Yoshihito Niki

**Affiliations:** Showa University School of Medicine, Tokyo, Japan (N. Hattori, T. Takuma, N. Arai, R. Yanai, S. Ishii, Y. Miura, T. Tokunaga, T. Nakamaki, Y. Niki);; National Institute of Infectious Diseases, Tokyo (M. Kuroda, H. Katano, T. Sekizuka, H. Hasegawa, T. Wakita);; Showa Uuniversity Koto Toyosu Hospital, Tokyo (T. Ito, H. Watanabe, N. Nomura, J. Eguchi)

**Keywords:** *Candidatus* Mycoplasma haemohominis, bacteria, mycoplasma, human infection, hemoplasma, hemophagocytic syndrome, metagenomics, Japan

## Abstract

Hemotropic mycoplasmas are common pathogens in animals, but it remains unclear what role these pathogens play in human infections. We report clinical and biologic characterization of *Candidatus* Mycoplasma haemohominis infection in a 42-year-old man in Japan. The patient had severe hemophagocytic syndrome 1 month after an accidental needlestick injury. Metagenomic deep sequencing identified *Candidatus* M. haemohominis and determined its draft genome for an isolate from serum of the patient. A high copy number of the *Candidatus* M. haemohominis genome was detected in serum and bone marrow samples. Electron microscopy examination showed morphologic characteristics of *Candidatus* M. haemohominis. Levofloxacin monotherapy induced resistance caused by a gyrase A gene mutation in the quinolone resistance–determining region, but a combination treatment with moxifloxacin and minocycline was effective. We identified *Candidatus* M. haemohominis in a patient who had life-threatening symptoms related to multiple organ infection. Human infection with this mycoplasma might occur more frequently than has been generally recognized.

Hemotropic mycoplasmas (or hemoplasmas; size <1 μm) are unculturable, cell wall–deficient, gram-negative bacteria that parasitize on the surface of the erythrocytes of numerous domestic and wild animals, such as cats, dogs, rodents, swine, cattle, sheep, bears, and bats (*1**–**4*). These pathogens can cause pyrexia, hemolytic anemia, and icterus (*1**,**5*). There have been few studies on molecular characterization of hemoplasmas to confirm infections in humans (*6**–**11*), possibly because hemoplasmas are unculturable and are liable to be overlooked by physicians (*11**,**12*). Steer et al. found that the putative species *Mycoplasma* species *Candidatus* Mycoplasma haemohominis can infect humans and cause hemolytic anemia and pyrexia (*9*). However, only a partial sequence of *Candidatus* M. haemohominis (GenBank accession no. GU562825) could be confirmed (*9*). Moreover, clinical manifestations of *Candidatus* M. haemohominis infections in humans have not been well characterized.

We identified and characterized *Candidatus* M. haemohominis infections in a patient with pyrexia of unknown origin. The patient had various life-threatening symptoms that were not limited to hemolytic anemia and was infected with this bacterium after an accidental needlestick injury. We also analyzed the genome of *Candidatus* M. haemohominis isolated from specimens obtained from the patient. This study was approved by the Institutional Review Board of Showa University (Tokyo, Japan) and the National Institute of Infectious Diseases (Tokyo). We obtained informed consent from the patient.

## Case-Patient

**Figure 1:**
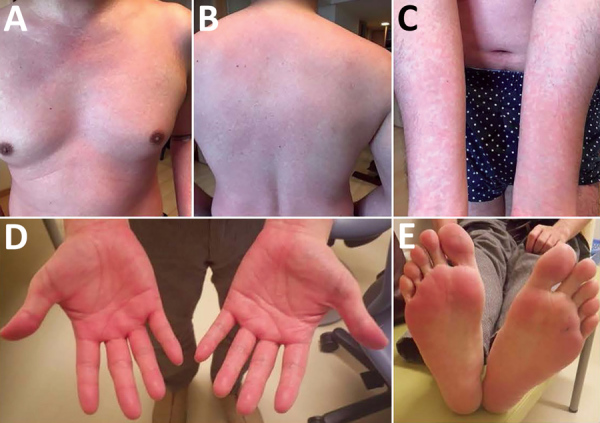
Whole-body erythema and pruritus in a 42-year-old man infected with *Candidatus* Mycoplasma haemohominis, Japan. Images show general erythema and pruritus covering >80% of the body surface area. A) Chest, B) back, C) arms, D) hands, E) feet.

The case-patient was a 42-year-old man (physician) who had no unusual medical history and no recent overseas travel history. The patient was admitted to Showa University School of Medicine Hospital (Tokyo, Japan) because of pyrexia, anemia, and liver dysfunction. One month before admission, he had accidentally pricked his finger when performing needle biopsy of the liver for 1 inpatient, who was admitted to this hospital because of cryptogenic liver injury and anemia after traveling overseas. Two weeks after the needlestick injury, the case-patient had pyrexia and whole-body erythema with pruritus (Figure 1). His rash disappeared after 3 days. However, he was admitted to the hospital because lymphadenopathy, hepatosplenomegaly, and pyrexia developed.

**Figure 2:**
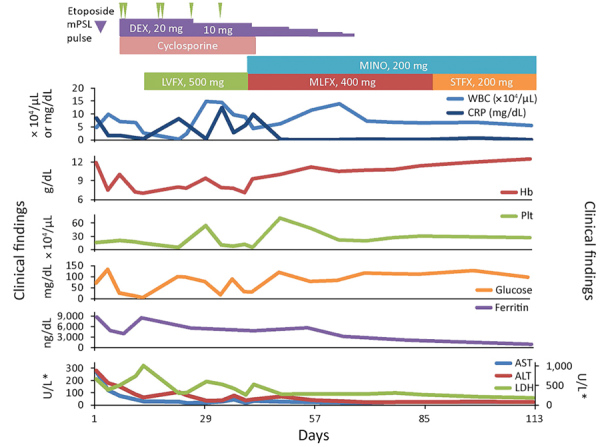
Clinical course for a 42-year-old man infected with *Candidatus* Mycoplasma haemohominis, Japan. *For ALT, AST, and LDH, left y-axis is for AST and ALT and right y-axis is for LDH. ALT, alanine aminotransferase; AST, aspartate aminotransferase; CRP, C-reactive protein; DEX, dexamethasone; Hb, hemoglobin; LDH, lactate dehydrogenase; LVFX, levofloxacin; MINO, minocycline; MLFX, moxifloxacin; mPSL, methylprednisolone; PLT, platelets; STFX, sitafloxacin; WBC, white blood cells.

We provide the clinical course for the case-patient (Figure 2). At admission, the case-patient had relative bradycardia (96 beats/min) and a body temperature of 39.5°C. Laboratory results showed an increase in levels of aspartate aminotransferase (274 U/L, reference range 10–40 U/L), lactate dehydrogenase (664 U/L, reference range 120–245 U/L), ferritin (8,748 ng/mL, reference range 20–400 ng/mL), soluble interleukin-2 receptor (8,791 U/mL, reference range 122–496 U/mL), and C-reactive protein (8.45 mg/dL, reference range 0.00–0.20 mg/dL). A complete blood count showed anemia (hemoglobin concentration 11.9 g/dL, reference range 13.6–18.3 g/L), but the leukocyte count (4.8 × 10^3^ cells/μL, reference range 3.5–9.0 × 10^3^ cells/μL) and platelet count (16.5 × 10^4^/μL, reference range 14.0–37.9 × 10^4^ cells/μL) were within reference intervals. A Coombs test result was negative, but low haptoglobin concentrations (<8 mg/dL, range 30–200 mg/dL) were found.

Test results were negative for hepatitis A, B, C, and E viruses; measles virus; rubella virus; parvovirus; and HIV. Results of antinuclear antibody (titer 1:80) and smooth muscle antibody (titer 1:40) tests were positive, but test results for antimitochondrial M2 antibody, mitochondrial antibody, double-stranded DNA antibody, and lupus anticoagulant were negative. Epstein-Barr virus (EBV) DNA load determined by PCR was 3.0 × 10^2^ copies/mL. However, Southern blot hybridization did not detect clonality of EBV-infected cells. Levels of herpes simplex virus, human herpesvirus 6 and 8, varicella zoster virus, and cytomegalovirus were below reference values, as determined by PCR. The serum IgG level was 3,967 mg/dL (reference range 800–1,750 mg/dL). However, the result of a serum-free light chain test was within the reference limit.

Our initial diagnosis was hemophagocytic syndrome (HPS) related to undetermined disease, and the patient was given steroid pulse therapy (1,000 mg) on day 1. Because his symptoms persisted, he was given etoposide (100 mg/m^2^), cyclosporine (5 mg/kg), and dexamethasone (20 mg) on day 7. After this treatment was started, whole-genome sequencing of a peripheral blood sample detected a novel hemotropic *Mycoplasma* sp.

**Figure 3:**
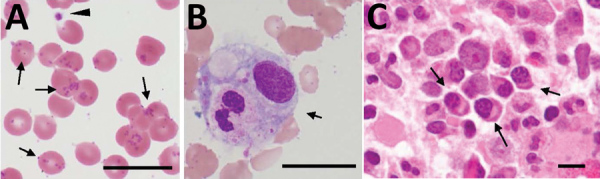
Distribution of *Candidatus* Mycoplasma haemohominis in a 42-year-old man, Japan. A) Peripheral blood smear showing coccoid forms. Small basophilic bodies are present on the surface of and outside erythrocytes (arrows). Arrowhead indicates a platelet. Giemsa stained. B) Hemophagocytosis (arrow) in bone marrow aspirate. Giemsa stained. C) Bone marrow biopsy specimen showing infiltration of plasma cells (arrows). Hematoxylin and eosin stained. Scale bars indicate 20 μm.

On day 11, we found a prolonged activated partial thromboplastin time (50.6 s, reference range 25–45 s), normal prothrombin time (12.6 s), low coagulation factor VIII activity (1.8%, reference range 78%–165%), and low von Willebrand factor (VWF) activity (ristocetin cofactor <10%, reference range 50%–150%). Moreover, serum levels of fibrinogen, antithrombin III, and disintegrin and metalloproteinase with thrombospondin type 1 motif, member 13 were within reference ranges. Microscopic examination of a Giemsa-stained blood smear showed coccoid forms on the erythrocyte surface (Figure 3, panel A). A bone marrow aspirate showed hemophagocytosis and increased levels of reactive plasma cells (19.5%) (Figure 3, panels B, C). On day 12, hypoglycemia (glucose level <5 mg/dL, reference range, 61–139 mg/dL) was noted in blood from the collection tube used for serologic tests, but the glucose level was within the reference range for blood in a container that contained citric acid with NaF.

On day 13, the case-patient received a diagnosis of *Candidatus* M. haemohominis infection–associated HPS and was given levofloxacin. Hypoglycemia improved after administration of levofloxacin. However, pyrexia and anemia developed, and coccoid forms on erythrocytes in peripheral blood and bone marrow were again observed on day 37. On day 46, he was given moxifloxacin and minocycline because of suspected bacterial resistance to levofloxacin. After this treatment was initiated, his symptoms, such as pyrexia, anemia, and hypoglycemia, promptly resolved, and the patient was discharged on day 61. One year after discontinuation of treatment, the patient remained well, and no *Candidatus* M. haemohominis DNA was detected in his serum.

## Materials and Methods

### High-Throughput Unbiased RNA Sequencing

We purified total RNA from a patient serum sample by using the RNeasy Mini Kit (QIAGEN, https://www.qiagen.com). We prepared RNA-Seq libraries by using the ScriptSeq version 2 RNA-Seq Library Preparation Kit (Illumina, https://www.illumina.com) and sequenced these libraries as single-end 151-mers by using the NextSeq 500 sequencer (Illumina) (*13*,*14*).

### Whole-Genome Analysis

We performed metagenomic short-read DNA sequencing by using NextSeq 500 (Illumina) with DNA extracted from serum specimens. We excluded human-related short reads by using a Burrows–Wheeler Mapping Program with default parameters against human genome sequences (GRCh38.p13) (*15*).

We obtained a draft genome sequence of *Candidatus* M. haemohominis by de novo assembly using metagenomic DNA-Seq short reads. First, we excluded human genomic DNA sequences (≈58.3%) by using read-mapping analysis, followed by de novo assembly with the remaining short reads. The total number of contigs was 703, and total length was ≈1.42 Mb, suggesting that whole contigs included bacterial and human-related DNA sequences. Therefore, to extract the bacterial sequences, we considered read depth and coverage, % GC of each contig, and a blastn search (https://blast.ncbi.nlm.nih.gov). A total of 23 contigs showed a marked read depth coverage (average × 5,500) and 30% GC content, although blastn search analysis showed that 4 contigs were assigned to the *Mycoplasma* genome and 19 contigs were assigned to unknown sequences.

### Electron Microscopy

We used negative staining for serum samples. Small aliquots of serum samples were absorbed onto glow-discharged, 300-mesh, heavy-duty carbon-coated copper Cu grids (Veco Grids; Nisshin EM, http://nisshin-em.co.jp) for 2 min, and excess liquid was blotted with Whatman filter paper (GE Healthcare, https:///www.gehealthcare.com). We then washed grids twice with Milli-Q water (http://emdmillipore.com) and negatively stained them with 2% uranyl acetate. We observed specimens by using an H7700 transmission electron microscope (Hitachi, https://www.hitachi.com) at 80 kV and ×10,000 magnification.

We fixed tissue samples with 2.5% glutaraldehyde and 2% paraformaldehyde in 0.1 mol/L phosphate buffer (pH 7.4) for 2 h at room temperature, postfixed these samples in 1% osmium tetroxide, and embedded them in Epon resin. We stained ultrathin sections with uranyl acetate and lead citrate and observed them under a transmission electron microscope (HT7700; Hitachi) at 80 kV.

### In Situ Hybridization

We used 16S and 23S rRNA genes for in situ hybridization analysis. We amplified a target fragment by using PCR with digoxigenin-11-dUTP and MHaemohominis1F (5′-AATTAACGCTGATGGCATGC-3′) and MHaemohominis600R (5′- TCCTACCGTATTCTAGACGGAC-3′) primers. We purified a PCR amplicon by using a PCR Purification Kit (QIAGEN) and denatured it by heat shock before hybridization. We treated deparaffinized slides with 0.3% H_2_O_2_/methanol for 30 min and 0.2 mol/L HCl for 20 min, then incubated slides with proteinase K (3–10 μg/mL) for 30 min at 37°C. After prehybridization, we hybridized slides with 2 pmol/L of denatured probe per slide in hybridization buffer (20% formamide; 5× saline–sodium citrate [SSC], 5 × Denhardt solution; 50 mmol/L HEPES buffer [pH 7.0], and 40 µg/mL salmon sperm DNA) overnight at 42°C. We washed the slides twice with 2× SSC at 50°C for 15 min and twice with 0.2× SSC at 50°C for 15 min, added anti-digoxigenin monoclonal antibody (Sigma, https://www.sigmaaldrich.com), and incubated the slides for 45 min. We than amplified signals by using a GenPoint Kit (Dako Agilent, https://www.agilent.com) and detected these signals by using 3,3′-diaminobenzidine as a chromogen.

### Real-Time PCR

We used a DNA fragment of the 16S rRNA gene of *Candidatus* M. haemohominis in a Taqman real-time PCR (*16*). We then performed PCR amplification in 25-μL reaction mixtures containing QuantiTect Probe PCR Master Mixture (QIAGEN), 0.4 μmol/L of each primer, 0.2 μmol/L of TaqMan probe, and 100 ng of isolated DNA. PCR conditions were 95°C for 15 min, followed by 45 cycles of 94°C for 15 s and 60°C for 1 min in a Mx3005P Apparatus (Dako Agilent).

### Sequence Data

We deposited metagenomic short-read sequences for DNA-Seq in the DDBJ (BioProject PRJDB7871; BioSample SAMD00156464; DRR accession no. DRR164892). We deposited the draft, annotated genome sequence of *Candidatus* M. haemohominis SWG34-3 in the DNA Data Bank of Japan (accession nos. SAMD00156495 and BIMN01000001–23). The 16S rRNA sequence was deposited under accession no. MHSWG343_r0010. The predicted gyrase *A* gene nucleotide sequence was deposited under accession no. MHSWG343_02220, and the coding sequence was deposited under accession no. GCE63237.1.

## Results

### Identification and Whole-Genome Sequencing of *Candidatus* M. haemohominis

**Figure 4:**
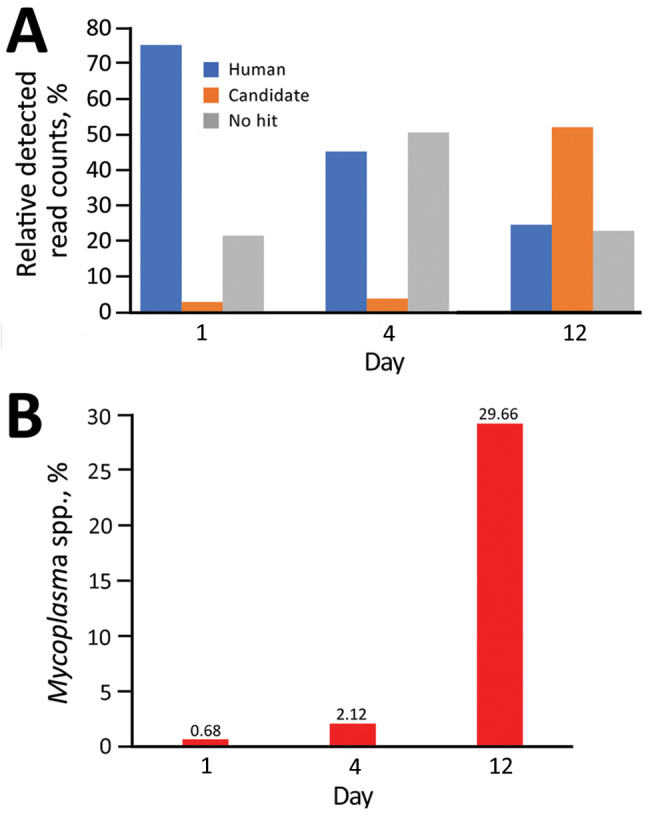
Prediction of presence of *Candidatus* Mycoplasma haemohominis in serum samples of a 42-year-old man, Japan. A) Relative percentage of candidate bacteria. B) Percentage of *Mycoplasma* spp. detected.

**Figure 5:**
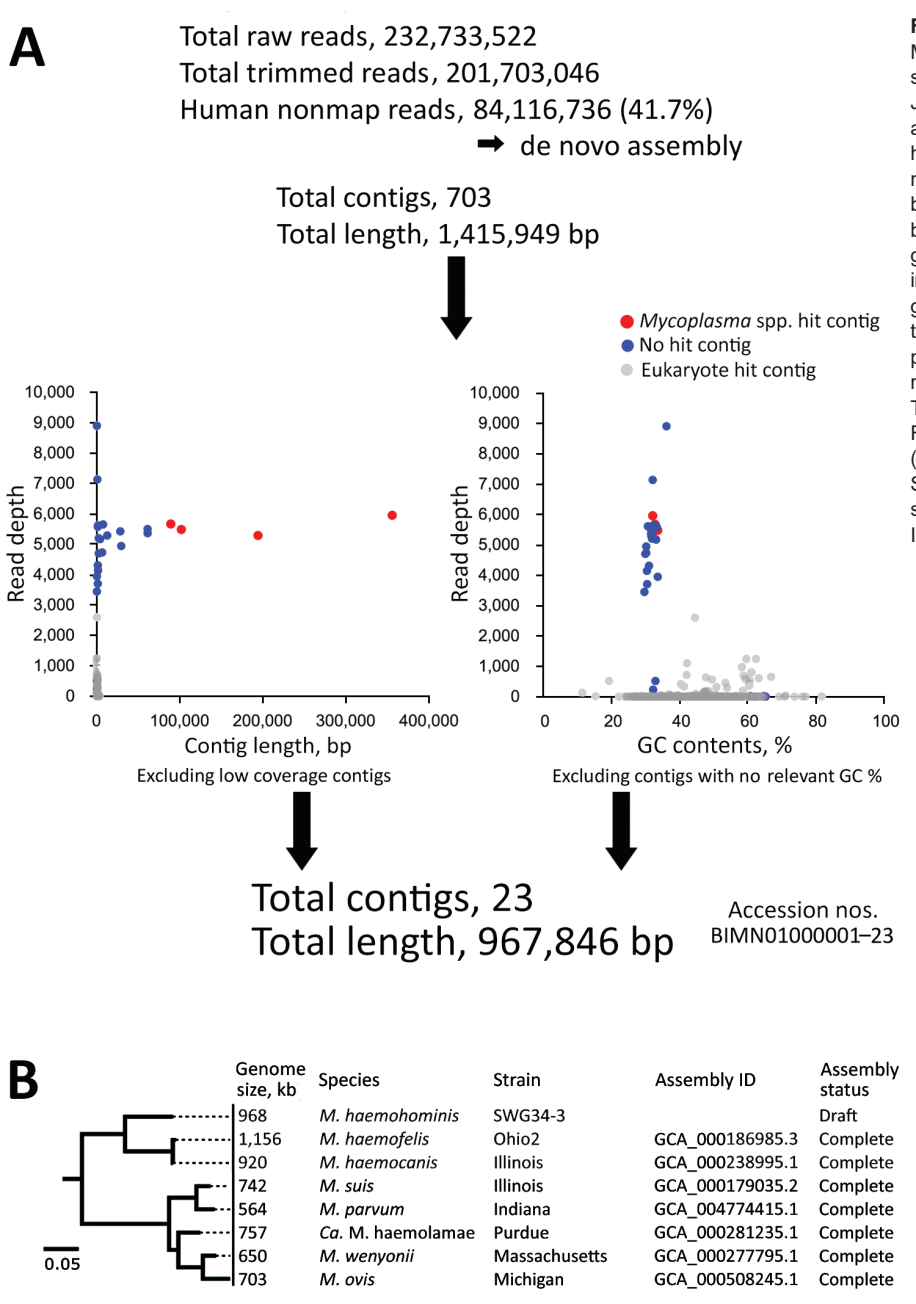
Analysis for *Candidatus* Mycoplasma haemohominis in serum of a 42-year-old man, Japan. A) Prediction that de novo assemblies contained bacteria and human DNA sequences. Bacteria-related sequences were identified by using read depth, % GC, and blastn (https://blast.ncbi.nlm.nih.gov) search results. Read depth indicates how many times next-generation sequencing confirmed the sequence at each nucleotide position. B) Phylogenetic tree of 16S rRNA genes of *Mycoplasma* spp. The tree was constructed by using FastTree version 2.1.10 (http://www.microbesonline.org). Scale bar indicates nucleotide substitutions per site. ID, identification.

Metagenomic deep RNA sequence analysis strongly suggested that rather than other pathogens or virus infections, the *Mycoplasma* spp. could be associated with signs and symptoms of the case-patient. RNA-Seq short reads related to the *Mycoplasma* spp. were increasingly detected in the serum of the case-patient (Figure 4). A total of 41.7% of human-unmapped reads were extracted and then subjected to de novo assembly to identify the mycoplasma draft genome (23 contigs, 967,846 bp) (Figure 5, panel A). The draft genome sequence identified a potential pathogen that could be *Candidatus* M. haemohominis on the basis of 16S rRNA sequence homology. Also, this pathogen showed similarity with closely related species, such as *M. haemofelis* and *M. haemocanis* (Figure 5, panel B).

**Figure 6:**
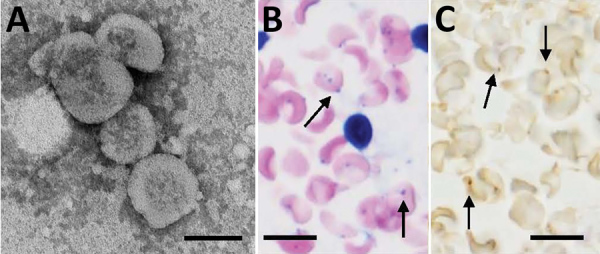
Morphologic features of *Candidatus* Mycoplasma haemohominis isolated from a serum sample of a 42-year-old man, Japan*.* A) Spheres are bacterial particles with a diameter of 300–600 nm. Negative stained; scale bar indicates 200 nm. B) Bacteria on the surface of erythrocytes (arrows in the left panel). In situ hybridization showing bacteria on the surface of erythrocytes (arrows in the right panel). Giemsa stained; scale bar indicates 10 μm.

Negative-staining and electron microscopy of *Candidatus* M. haemohominis particles identified ribosomes, DNA, and soluble RNA in the cytoplasm (Figure 6, panel A). In situ hybridization identified *Candidatus* M. haemohominis on the surface of erythrocytes and in the cytoplasm of macrophages in bone marrow (Figure 6, panel B).

### Monitoring and Treatment for *Candidatus* M. haemohominis Infection

**Figure 7:**
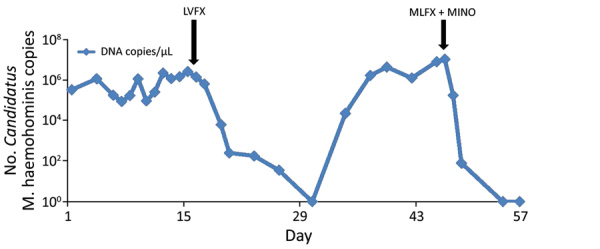
Copy number of the *Candidatus* Mycoplasma haemohominis genome in 1-μL serum samples from a 42-year-old man, Japan. Copy number was determined by using a real-time PCR. LVFX, levofloxacin; MINO, minocycline; MLFX, moxifloxacin.

**Figure 8:**
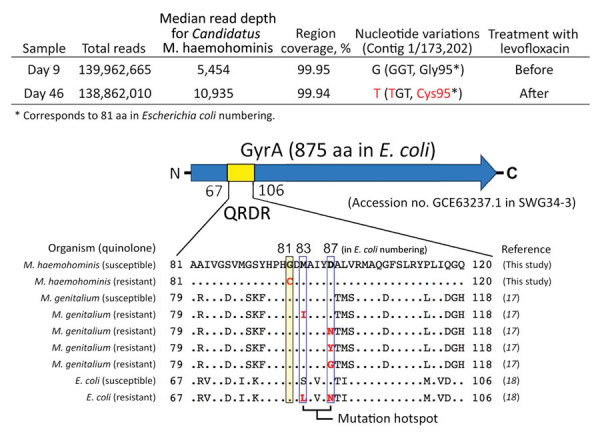
Detection of a *gyrA* mutation in the QFDR of *Candidatus* Mycoplasma haemohominis genome isolated from in a 42-year-old man, Japan, who was given levofloxacin. Read-mapping analysis identified a nonsynonymous amino acid substitution in gyrA for quinolone-resistant *Candidatus* M. haemohominis from a serum sample. Bottom panel shows a schematic of the gyrA amino acid sequence and alignment with those of other QRDRs (*17**,**18*). Dots indicate identical amino acids corresponding to the *Candidatus* M. haemohominis sequence. Red indicates nucleotide or amino acid mutations. Arrow indicates direction of transcription. QRDR, quinolone resistance–determining region.

We determined the level of *Candidatus* M. haemohominis DNA in serum by using real-time PCR (Figure 7). A high copy number for *Candidatus* M. haemohominis DNA was detected 18 days after the accidental needlestick injury. The bacterial load in serum decreased below the detection limit (<10 copies/reaction) 14 days after the patient was given levofloxacin, but again increased. Metagenomic analysis also indicated a high detection rate for *Mycoplasma* spp. reads in serum before and after treatment with levofloxacin. Read-mapping analysis showed that only 2-nt mutations were identified in samples after treatment with levofloxacin. Moreover, 1 of 2 mutations was a nonsynonymous mutation in the quinolone resistance–determining regions (QRDR) of the DNA gyrase subunit A GyrA (i.e., Gly95Cys, corresponding to aa 81 by numbering for *Escherichia coli*) (Figure 8). The bacterial load promptly decreased after combination therapy with moxifloxacin and minomycin. We also identified *Candidatus* M. haemohominis by using real-time PCR and in situ hybridization with samples from a liver biopsy of the patient.

## Discussion

Infection with *Candidatus* M. haemohominis needs to be distinguished from other hemoplasmas in terms of clinical symptoms. As described previously, infection with possible hemoplasmas results in hemolytic anemia and pyrexia (*6**–**11*). Our results showed that infection with *Candidatus* M. haemohominis can cause various life-threatening symptoms in humans, such HPS, liver damage, and bleeding.

We identified *Candidatus* M. haemohominis in the case-patient and clarified the clinical features of *Candidatus* M. haemohominis infection as follows. First, *Candidatus* M. haemohominis infection might cause HPS. Second, this infection might cause reactive plasmacytosis. Third, the increase in plasma cells might induce antibody production and hypergammaglobulinemia. Fourth, infected patients might have coccoid bacterial forms on erythrocyte surfaces and pseudohypoglycemia in vitro. Fifth, this infection might also be accompanied by mild to severe hemorrhagic episodes.

We suggest that our results will help in diagnoses of *Candidatus* M. haemohominis infections, enabling early therapeutic intervention that might cure patients with these infections. *Candidatus* M. haemohominis patients are highly susceptible to misdiagnosis with other diseases, such as collagen diseases (e.g., systemic lupus erythematosus) and virus infections (e.g., severe fever with thrombocytopenia syndrome and EBV-associated HPS), because of the wide range of complications.

Generally, antimicrobial drug therapy with fluoroquinolones or tetracyclines is effective against hemoplasmas in animals. Combination therapy (*9*) or sequential treatment (*19*) with these drugs can be more effective against hemoplasmas because a single agent is often insufficient for consistent elimination of bacteremia (*19*). Moreover, detection of mutations in QRDR of gyrase A (Gly95Cys) suggested that *Candidatus* M. haemohominis is resistant to older fluoroquinolones (e.g., levofloxacin) (*20**,**21*). In contrast, newer fluoroquinolones, such as moxifloxacin or sitafloxacin, and tetracyclines, are effective against mycoplasmas harboring this mutation (*21*).

Laboratory tests for our case-patient showed low blood glucose levels (<5 mg/dL, reference range 61–139 mg/dL) but related no clinical signs. Hemoplasma-associated hypoglycemia in the absence of any associated clinical signs has been described in various animal species (*22*). This phenomenon is proportional to the severity of bacteremia and depends on the hemoplasma species (*22*). Because it is possible that glucose would be useful only for carbohydrate metabolism in the hemoplasma species (*23*), this finding might cause attachment in the epierythrocytic environment, which is rich in glucose, and result in alternative energy source pathways that become redundant.

We observed moderate to severe hemorrhagic episodes for this case-patient. Although the pathogenesis of bleeding in patients infected with *Candidatus* M. haemohominis is not completely understood, a dramatic decrease in coagulation factor VIII and VWF activities was observed in this case-patient, which is similar to that observed in patients with acquired von Willebrand syndrome (*24**,**25*). Given the hypergammaglobulinemia and increased plasma cell levels observed in this case-patient and other patients. the possible mechanisms of infection are adsorption of vWF onto plasma cells or activated platelets apart from the presence of vWF-specific antibodies and inhibitors (*24**,**25*).

In conclusion, we identified and characterized *Candidatus* M. haemohominis infection in a human. Although our study has limitations, our results provide useful knowledge about this infection. Other patients infected with *Candidatus* M. haemohominis may have died before a confirmed diagnosis was made. Thus, *Candidatus* M. haemohominis infection might occur more frequently than generally recognized. Further epidemiologic investigations of infection with *Candidatus* M. haemohominis in humans and of intermediate host(s) for this pathogen would clarify the extent of human infection and possible transmission routes.
